# Pancreatic cancer-derived exosomal microRNA-19a induces β-cell dysfunction by targeting ADCY1 and EPAC2

**DOI:** 10.7150/ijbs.56271

**Published:** 2021-08-21

**Authors:** Wenjing Pang, Weiyan Yao, Xin Dai, Aisen Zhang, Lidan Hou, Lei Wang, Yu Wang, Xin Huang, Xiangjun Meng, Lei Li

**Affiliations:** 1 Department of Gastroenterology, Shanghai Jiaotong University School of Medicine affiliating Shanghai 9th People's Hospital, Shanghai, China.; 2 Department of Gastroenterology, Shanghai Jiaotong University School of Medicine affiliating Shanghai Ruijin Hospital, Shanghai, China.; 3 Jiangsu Engineering Research Center for microRNA Biology and Biotechnology, State Key Laboratory of Pharmaceutical, Biotechnology, School of Life Sciences, Nanjing University, Nanjing, China.; 4 Department of Gerontology, Jiangsu People's Hospital affiliating to Nanjing Medical University, Nanjing, China.; 5 Digestive Disease Research and Clinical Translation Center, Shanghai Jiaotong University, Shanghai, China.

**Keywords:** β cell dysfunction, miR-19a, exosome, pancreatic neoplasm

## Abstract

New-onset diabetes mellitus has a rough correlation with pancreatic cancer (PaC), but the underlying mechanism remains unclear. This study aimed to explore the exosomal microRNAs and their potential role in PaC-induced β-cell dysfunction. The pancreatic β cells were treated with isolated exosomes from PaC cell lines, SW1990 and BxPC-3, before measuring the glucose-stimulated insulin secretion (GSIS), validating that SW1990 and BxPC-3 might disrupt GSIS of both β cell line MIN6 and primary mouse pancreatic islets. The difference in expression profiles between exosomes and exosome-free medium of PaC cell lines was further defined, revealing that miR-19a secreted by PaC cells might be an important signaling molecule in this process. Furthermore, adenylyl cyclase 1 (Adcy1) and exchange protein directly activated by cAMP 2 (Epac2) were verified as the direct targets of exogenous miR-19a, which was involved in insulin secretion. These results indicated that exosomes might be an important mediator in the pathogenesis of PaC-DM, and miR-19a might be the effector molecule. The findings shed light on the pathogenesis of PaC-DM.

## Introduction

Numerous epidemiological studies have investigated the correlation between pancreatic cancer (PaC) and diabetes mellitus (DM) 1-3. More than 80% of patients with PaC have glycometabolic disorders within 3 years before the diagnosis of cancer 4, 5. A recent meta-analysis including a total of 35 cohort studies support that diabetes is associated with an increased risk of pancreatic cancer in both males and females and that DM has a relative risk of pancreatic cancer negatively correlated with the duration of DM, which is highest within less than 1 year 6. Thus, DM is an early symptom induced by PaC although it may be an established risk factor for cancer development. However, PaC-associated diabetes (PaC-DM) differs from type 1 DM or type 2 DM. According to the classification of diabetes mellitus established by American Diabetes Association (ADA) 7, it belongs to type 3c diabetes mellitus (T3cDM) that is caused by inherited or acquired pancreatic exocrine disease, whose characteristics are hypoinsulinemia, lack of pancreatic polypeptide, and hepatic insulin resistance, etc.

However, how cancer influences metabolism is still unclear. Evidences showed that some tumor-mediated factors, such as adrenomedullin 8, might induce insulin resistance and β-cell dysfunction in PaC-DM. Exosomes are tiny vesicles produced from the plasma membrane in physiological or pathological status, having a diameter of 30-150 nm 9. Given that microRNAs (miRNAs) can repress the protein level by accelerating messenger RNA degradation and inhibiting translation 10, emerging studies demonstrated that exosomes could transport secreted miRNAs to target cells and modulate biological functions by downregulating the expression of target molecules 11-13. Interestingly, mounting evidences have shown the changes of microRNA profile in PaC 14. Considering the fact that tumor cells express certain oncogenic miRNAs and can generate and release high levels of miRNA-containing exosomes 15, we hypothesized that PaC cells might actively induce β-cell dysfunction by delivering tumor-specific miRNAs via an exosome-based pathway.

In a previous study of our group, 16 serum miRNAs, whose expression significantly increased in the PaC-DM samples, were identified. A combination of six serum miRNAs (miR-483-5p, miR-19a, miR-29a, miR-20a, miR-24, and miR-25) was selected by qRT-PCR as a biomarker for PaC-DM 16. Based on these results, we tried to figure out the potential miRNAs in this panel that might mediate the PaC-induced glycometabolic disorder. In the present study, we investigated the involvement of PaC cells exosomal miRNAs on the impact of islets β cells dysfunction. We revealed that exosomal miR-19a, which was released by pancreatic cancer cells, incorporated by MIN6 and isolated mouse islets, could dramatically damage the GSIS. Furthermore, the direct target genes inducing insulin exocytosis in pancreatic β cells were confirmed using bioinformatic analysis. Our work not only replenishes studies on the nosogenesis of this cancer-related disorder but also help explore potential tools for PaC screening in patients with new-onset DM.

## Materials and Methods

### Pancreatic cell lines, primary pancreatic islets, and culture conditions

Human PaC cell lines SW1990 and BxPC-3 were purchased from the Shanghai Institute of Cell Biology, Chinese Academy of Sciences (Shanghai, China), and the mouse PaC cell line PAN02 was purchased from the Shanghai Fu Heng Biology (Shanghai, China). All the cells were cultured in Dulbecco's Modified Eagle Medium (DMEM) supplemented with 10% Fetal Bovine Serum (FBS, Gibco) and were grown in a humidified 5% CO_2_ atmosphere with a temperature of 37 °C.

Mouse pancreatic β cell line MIN6 (obtained from Jiangsu Engineering Research Center for MicroRNA Biology) cells were cultured in Roswell Park Memorial Institute (RPMI) 1640 supplemented with 15% FBS and 100 µmol/L 2-mercaptoethanol (Sigma, M3148). Primary mouse islets were isolated from wild-type male 6~8 week-old C57BL/6J mice by collagenase V (Sigma) digestion and Histopaque (Sigma) as described previously 17, hand-picked, and cultured, prior to further investigations, for at least 12 h in RPMI 1640 medium containing 10 mmol/L glucose, 15% FBS, 100 U/mL penicillin, and 100 μg/mL streptomycin. Both MIN6 cells and primary pancreatic islets were grown in a humidified 5% CO_2_ atmosphere with a temperature of 37 °C.

### Measurement of glucose-stimulated insulin secretion

The glucose-stimulated insulin secretion (GSIS) was measured as described previously 18, and the detail was described in the Supplementary Methods. The levels of insulin were measured using a rat/mouse insulin ELISA kit (EZRMI-13K, Millipore, Germany).

### Exosome isolation

Exosomes were isolated from cell culture supernatant (after 48-h cultivation) using a commercial total exosome isolation kit (Umibio, Shanghai, China; Cat No. UR52101) following the manufacturer's protocols. The exosome pellet part was then resuspended in a volume of RNAase-free water equal to the starting volume of the biofluid to determine the relative abundance of specific exosomal or non-exosomal miRNAs in PaC cell supernatants.

The bicinchonininc acid (BCA) method was used to quantify the total protein content in the isolated exosomes before storage at -80 °C for further use.

### Transmission electron microscopy

Isolated cell-secreted vesicle preparations were fixed in 2.5% paraformaldehyde for 30 min and 2% osmium tetroxide in 0.1 M cacodylate buffer for 2 h sequentially at room temperature. Then, the pellets were dehydrated through a graded series of ethanol, rinsed with acetone twice, and embedded in Poly/Bed 812 (Polysciences Inc., USA). These tissue blocks were polymerized at 65 °C for 48 h before staining with uranyl acetate for 10 min and lead acetate for 10 min. A Hitachi-HT7700 transmission electron microscope (TEM) was used for the examination.

### Fluorescence labeling of exosomes

Fluorescence labeling of exosomes was performed as described previously 19, and the detail was described in the Supplementary Methods.

### Cell transfection with ncRNA, anti-miR-19a, or pre-miR-19a

The cells were seeded on six-well plates and transfected the following day using Lipofectamine 2000 (Invitrogen, CA) based on the manufacturer's protocols. For each well, an equal dose (100 pmol) of pre-miR-control, pre-miR-19a, anti-miR-control, or anti-miR-19a (GenePharma, China) was added. The cell culture supernatants or the cells were harvested 48 h after transfection for further experiments.

A green fluorescence protein (GFP)-labeled miR-19 inhibitor lentivirus was purchased (Asia-Vector Biotech., China) and transfected into PAN02 cells according to the manufacturer's instructions for further homograft use.

### RNA isolation and quantitative Real Time-Polymerase Chain Reaction (RT-PCR) of various mRNAs and miRNAs

The total RNA of exosomes derived from cancer cells or MIN6 cells was extracted using TRIzol Reagent (Invitrogen). Quantitative RT-PCR was carried out using TaqMan miRNA probes (Applied Biosystems, CA, USA) or the SYBR Green dye (Invitrogen) and specific primers of the targets following the manufacturer's protocols. The sequences of the primers used in this study are listed in Supplementary Table 1.

### Protein isolation and Western blot analysis

The cells were lysed in a RIPA lysis buffer. The supernatant was collected, and the protein concentration was calculated with a BCA protein assay kit (Thermo Scientific, USA). ADCY1, EPAC2, and GAPDH antibodies were purchased from Santa Cruz Biotechnology (sc-25743, sc-28326, and sc-32233, respectively). CD63, CD81, CD9, and TSG101 antibodies were purchased from Abcam (Cambridgeshire, UK, ab21735, ab109201, ab92726, and ab125011, respectively).

### Plasmid construction and luciferase assay

Luciferase reporter assays were performed as previously described 20 to test the direct binding of miR-19a to the target gene *Adcy1* and* Epac2*, and the detail was described in the Supplementary Methods.

### Glucose-induced Ca^2+^ oscillation imaging and measurement of cAMP levels in cultured β-cells

A Ca^2+^-selective fluorescent indicator, Fura-2/AM, was purchased from Abcam (ab120873), and the [Ca^2+^] concentration was measured using dual-wavelength fluorometry as described previously 21. The cytoplasmic cAMP levels were measured using a commercial cAMP Assay Kit (Abcam, AB65355) following the manufacturer's protocols.

### Establishment of tumor homograft in mice, insulin tolerance test, glucose tolerance test and *in vivo* GSIS

Four-week-old male nude mice were maintained under specific pathogen-free conditions. PAN02 cells were orthotopically injected into the pancreas of nude mice, and further assays were effectuated 30 days after homotransplantation. For glucose tolerance tests and *in vivo* GSIS assessment, a glucose solution (2 g/kg) was injected intraperitoneally into the mice after an overnight fast. Blood glucose was monitored using a glucometer system at the indicated times. Serum was collected at different time points from tails and insulin was measured. For insulin tolerance tests, an insulin solution (0.75I U/kg) was injected intraperitoneally into the mice after an overnight fast. Blood glucose was monitored using a glucometer system at the indicated times. After the sacrifice of mice, the orthotopic tumors and pancreatic islets were isolated and collected for further investigation. All animal care and handling procedures were performed in accordance with the National Institutes of Health's Guide for the Care and Use of Laboratory Animals and were approved by the Institutional Animal Care and Use Committee of Shanghai Ninth People's Hospital, School of Medicine, Shanghai Jiaotong University (Shanghai, China).

### Statistical analysis

The Western blot analysis of images was performed using ImageJ software and was representative of at least three independent experiments. Quantitative RT-PCR was performed in triplicate. The results were presented as the mean ± standard deviation of three independent experiments. The differences were considered statistically significant at *P* <0.05 using the Student *t* test.

## Results

### Disrupted GSIS by cell culture medium or isolated exosomes from PaC cell lines

**Figure 1:**
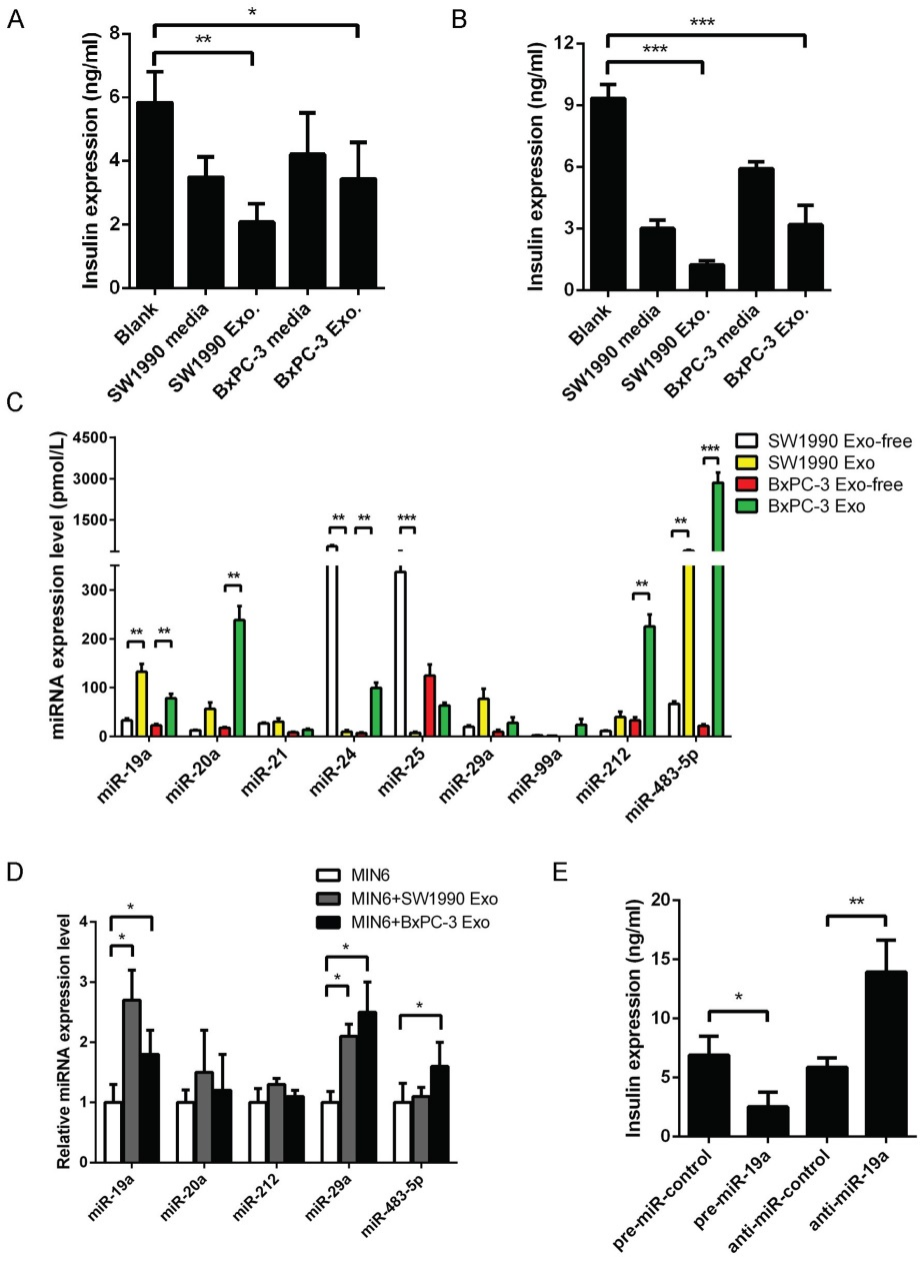
** (A)** GSIS assay in supernatants or exosomes derived from PaC treated-MIN6. **(B)** GSIS assay in supernatants or exosomes derived from PaC treated-primary islets. **(C)** Quantitative RT-PCR analysis of miRNA expression levels in exosomes and exosome-free supernatant of PaC cells. **(D)** Relative expression level of miRNAs in MIN6 cells treated with PaC cell exosomes. **(E)** Impact of miR-19a on the GSIS assay in MIN6 cells. *P < 0.05; **P < 0.01; ***P < 0.001.

Pancreatic cancer-derived exosomes were purified from the supernatants of cell lines SW1990 and BxPC-3, and validated using TEM (Supplementary Fig. 1A), NTA (Supplementary Fig. 1B), and exosomal marker protein (Supplementary Fig. 1C). As the glycometabolic disorders usually appeared before the tumor mass could be detected, it was supposed that the effector should be secreted outside PaC cells to induce intercellular communication and was mediated by exosomes, most likely, via the endocytic pathway. When Dil-C16-labeled vesicles, collected from supernatants of SW1990 and BxPC-3 cell lines, were added to MIN6 cells, the fluorescence was obviously observed in MIN6 cells after 6-h cultivation (Supplementary Fig. 1D), confirming that exosomes from human pancreatic cancer cell lines were incorporated by pancreatic islet cells. The supernatant of PaC cell lines collected after 48-h cultivation was used to replace MIN6 cell culture medium completely. In the meantime, the PaC-derived exosomes equivalent to 100 µg total protein were also added to MIN6 cells without changing 15% RPMI 1640 medium. These stimulated MIN6 cells were cultured for another 24 h before performing the GSIS. The GSIS of MIN6 cells treated with PaC supernatant showed a decline, while insulin secretion in 28 mmol/L glucose medium significantly altered in PaC exosome-treated β cells, compared with the control group (Fig. 1A). Further, the same experiments were performed in prepared primary islets (Supplementary Fig. 1E), revealing that the GSIS of islets was impaired by PaC supernatant and especially obvious using PaC-derived exosomes (Fig. 1B). These findings suggested that PaC-secreting exosomes might be one of the important pathways inducing β-cell dysfunction.

### Identification of miR-19a as the direct effector molecule in PaC-derived exosomes

The selection of six serum miRNAs (miR-483-5p, miR-19a, miR-29a, miR-20a, miR-24, and miR-25) as biomarkers for PaC-DM 16 by our group were supposed to be possible mediators in the GSIS defect. The present study evaluated the relative expression levels of these miRNAs in exosomes or exosome-free fractions of PaC cell supernatants. A profile of miR-19a, miR-20a, miR-29a, and miR-483-5p was primarily localized in the exosomal fraction, while miR-24 and miR-25 were localized in the exosome-free fraction (Fig. 1C). Subsequently, MIN6 cells were treated with pancreatic cancer cell-derived exosomes for 24 h, and changes in related miRNAs were defined. The study focused on miR-19a, considering that a dramatic upregulation of miR-19a was seen in exosome-treated MIN6 cells (Fig. 1D).

Our study found a high abundance of miR-19a in PaC-derived exosomes. However, whether the disturbance of miR-19a itself in MIN6 cells disrupted the GSIS remained unclear. Thus, miR-19a was overexpressed or knocked down by transfection into MIN6 cells and mouse primary pancreatic islets, indicating that the GSIS could be dramatically inhibited by exogenous miR-19a (Fig. 1E). Besides, the study also tested the pre-miR-19a mRNA levels in MIN6 treated with PaC cell supernatants and exosomes, revealing that the levels of endogenous miR-19a did not vividly increase with exogenous PaC stimulation (Supplementary Fig. 2A). Hence, it was speculated that PaC cells secreted exosomes actively to induce pancreatic β-cell dysfunction, and exosome-packed miR-19a might be the mediator.

### Prediction of *Adcy1* and *Epac2* as direct targets of miR-19a

**Figure 2:**
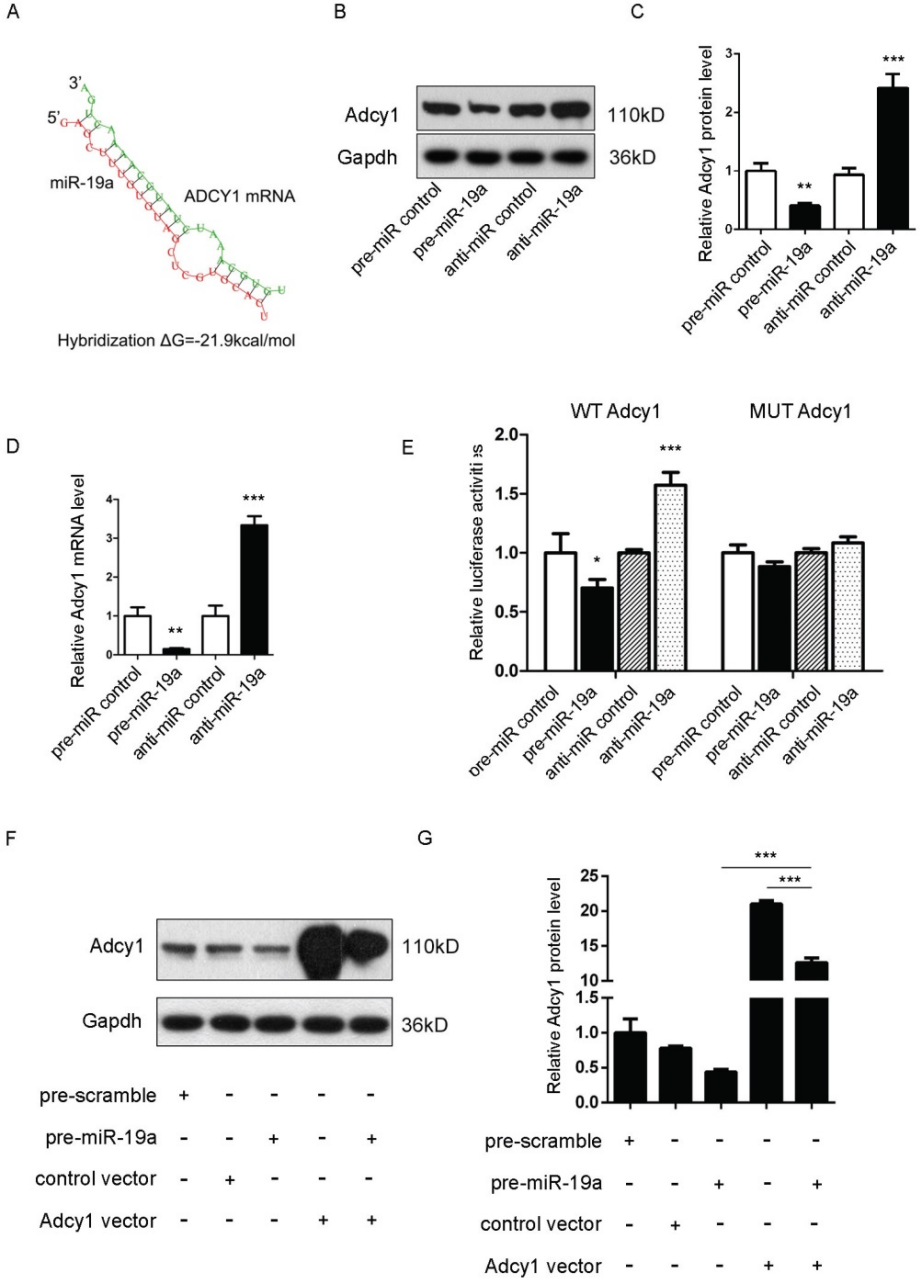
** (A)** Predicted free energy value of hybrid of Adcy1 3'-UTR and miR-19a. **(B and C)** Western blot analysis of Adcy1 protein levels in MIN6 cells treated with pre-miR-19a or anti-miR-19a. (B) Representative image. (C) Quantitative analysis. **(D)** Quantitative RT-PCR analysis of Adcy1 mRNA levels in MIN6 cells treated with pre-miR-19a or anti-miR-19a. **(E)** Luciferase reporters containing wild-type (WT) or mutant (MUT) miR-19a-binding sites in Adcy1 3'-UTR were co-transfected into MIN6 cells with pre-miR-19a or anti-miR-19a. The results were calculated as the ratio of luciferase activity in pre-miR-19a- or anti-miR-19a-transfected cells normalized to control cells. **(F and G)** Adcy1 overexpression plasmids were transfected into MIN6 cells with pre-miR-19a or pre-miR-control. The results were calculated as the ratio of protein expression levels normalized to corresponding controls. *P < 0.05; **P < 0.01; ***P < 0.001.

**Figure 3:**
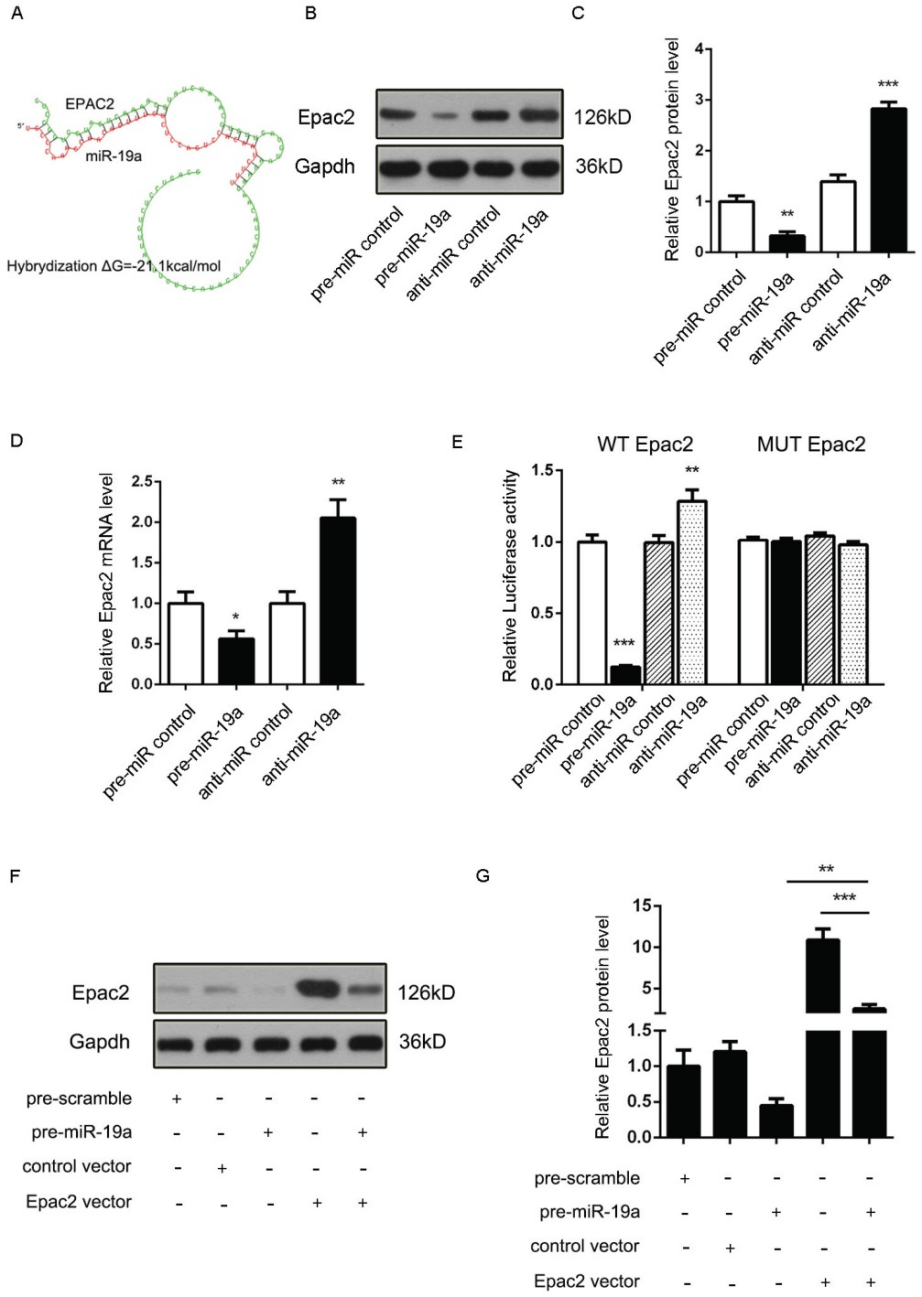
** (A)** Predicted free energy value of the hybrid of Epac2 3'-UTR and miR-19a. **(B and C)** Western blot analysis of Epac2 protein levels in MIN6 cells treated with pre-miR-19a or anti-miR-19a. (B) Representative image. (C) Quantitative analysis. **(D)** Quantitative RT-PCR analysis of Epac2 mRNA levels in MIN6 cells treated with pre-miR-19a or anti-miR-19a. **(E)** Luciferase reporters containing wild-type (WT) or mutant (MUT) miR-19a-binding sites in the Epac2 3'-UTR were co-transfected into MIN6 cells with pre-miR-19a or anti-miR-19a. The results were calculated as the ratio of luciferase activity in pre-miR-19a- or anti-miR-19a-transfected cells normalized to control cells. **(F and G)** Epac2 overexpression plasmids were transfected into MIN6 cells with pre-miR-19a or pre-miR-control. The results were calculated as the ratio of protein expression levels normalized to corresponding controls. *P < 0.05; **P < 0.01; ***P < 0.001.

It was proposed that miR-19a might be an important signaling molecule. However, target genes directly induced in β cells remained to be defined. Three computational algorithms, including TargetScan 22, PicTar 23, and MiRanda 24, were used in combination to identify the potential targets of miR-19a. *Adcy1 and Epac2*, implicated in insulin exocytosis, were predicted to be direct targets; the minimum free energy value of these hybrids 25 was -21.9 and -21.1 kcal/mol, respectively (Fig. 2A and 3A).

Adcy1 protein and mRNA levels were downregulated after the transfection of pre-miR-19a into MIN6 cells and upregulated by anti-miR-19a (Fig. 2B-D). The constructed plasmid containing presumed conserved binding sites of *Adcy1* fused downstream of the firefly luciferase gene, along with pre-miR-19a or anti-miR-19a and control plasmid (β-gal), was transfected into MIN6 cells to determine whether the negative regulatory effects of miR-19a on target protein expression were mediated through the binding of presumed sites in the 3'UTR of mRNA. As expected, the overexpression of miR-19a resulted in a reduction of luciferase reporter activity, while the decreased expression of miR-19a caused augmentation of luciferase reporter activity (Fig. 2E). Then, point mutations were introduced into the corresponding complementary sites to eliminate the predicted miR-19a-binding sites (Supplementary Fig. 2B; all the examined binding positions were mutant). These mutated luciferase reporters were unaffected by the overexpression or knockdown of miR-19a (Fig. 2E). This finding suggested that the binding sites strongly contributed to miRNA-mRNA regulation. Moreover, the cells transfected with the *Adcy1*-overexpressing plasmid could partially recuperate the Adcy1 protein level compared with the cells transfected with pre-miR-19a (Fig. 2F and 2G).

Accordingly, a similar study demonstrated that the Epac2 mRNA and protein levels might be downregulated by pre-miR-19a and upregulated by anti-miR-19a (Fig. 3B-D). Likewise, the potential role of the Epac2 mRNA 3'-UTR-binding sites in the regulation by miR-19a was also verified by the luciferase assay (Fig. 3E). Moreover, the decreased Epac2 protein level by pre-miR-19a could be recovered by the transfection of the corresponding overexpression plasmid (Fig. 3F and 3G). These results strongly suggested that the *Adcy1* and *Epac2* genes might be the direct targets of miR-19a.

### Role of secreted miR-19a and PaC cell-derived exosomes in the alteration of the expression of Adcy1 and Epac2 in β cells

**Figure 4:**
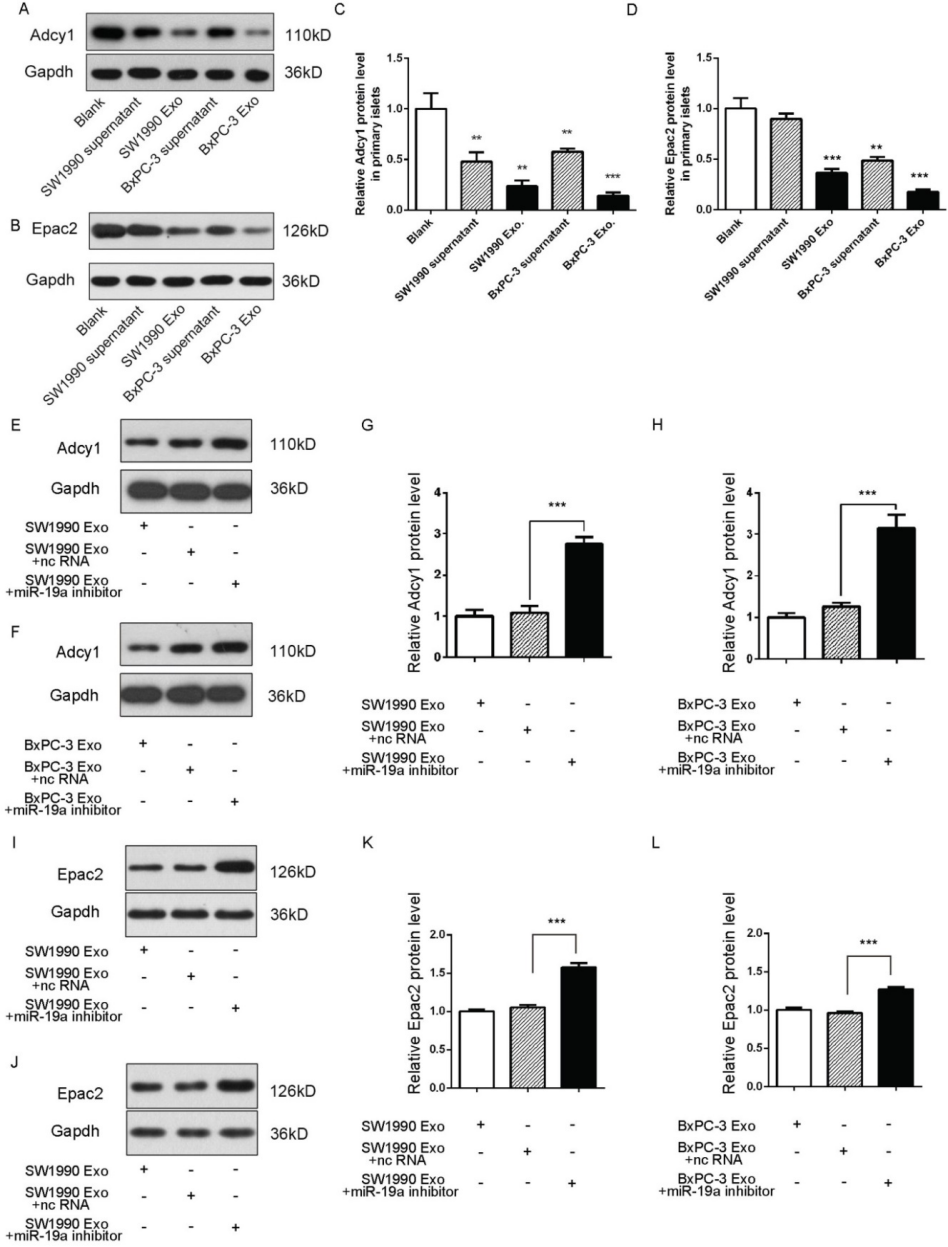
** (A-D)** Western blot analysis of Adcy1 and Epac2 protein levels in primary islets treated with exosomes derived from PaC cells. (A and B) Representative image. (C and D) Quantitative analysis. **(E-H)** Western blot analysis of Adcy1 protein levels in islets treated with miR-19a-deficient exosomes from SW1990 (E and G) or BxPC-3 (F and H) cells and controls. (E and F) Representative image. (G and H) Quantitative analysis. **(I-L)** Western blot analysis of Epac2 protein levels in MIN6 cells treated with miR-19a-deficient exosomes from SW1990 (I and K) or BxPC-3 (J and L) cells and controls. I and J: Representative image; K and L: quantitative analysis. *P < 0.05; **P < 0.01; ***P < 0.001.

Exosomes deliver miRNAs into recipient cells; these exogenous miRNAs can silence their target genes and trigger downstream signaling events. Hence, it was hypothesized that miR-19a was secreted from PaC cells into circulation in a stable and cell-free form and influenced β cells by targeting Adcy1 and Epac2. The SW1990 and BxPC-3 cell supernatants and exosomes were collected and used to treat primary islets. As expected, the Adcy1 and Epac2 protein levels dramatically decreased in PaC-derived exosome groups (Fig. 4A-D).

Exosomes containing various substances were shown to target recipient cells for the exchange of RNAs, proteins, and lipids 26, 27. Therefore, this study next tested whether miR-19a-deficient exosomes could induce alteration in the levels of Adcy1 and Epac2 proteins. SW1990 and BxPC-3 cells were transfected with anti-miR-19a, and exosomes were isolated from cell supernatants after 48-h cultivation. The same cell lines transfected with anti-ncRNA served as negative controls. The knockdown efficiency of anti-miR-19a in exosomes is shown in Supplementary Fig. 2C. The primary islets treated with miR-19a-deficient exosomes showed no remarkable changes in the protein levels of Adcy1 (Fig. 4E-H) and Epac2 (Fig. 4I-L). Consequently, it was concluded that miR-19a packaged in exosomes derived from PaC cells predominantly participated in the suppression of Adcy1 and Epac2 proteins related to insulin secretion.

### Changes in insulin exocytosis induced by PaC-derived exosomal miR-19a and the target *Adcy1* and *Epac2* genes

**Figure 5:**
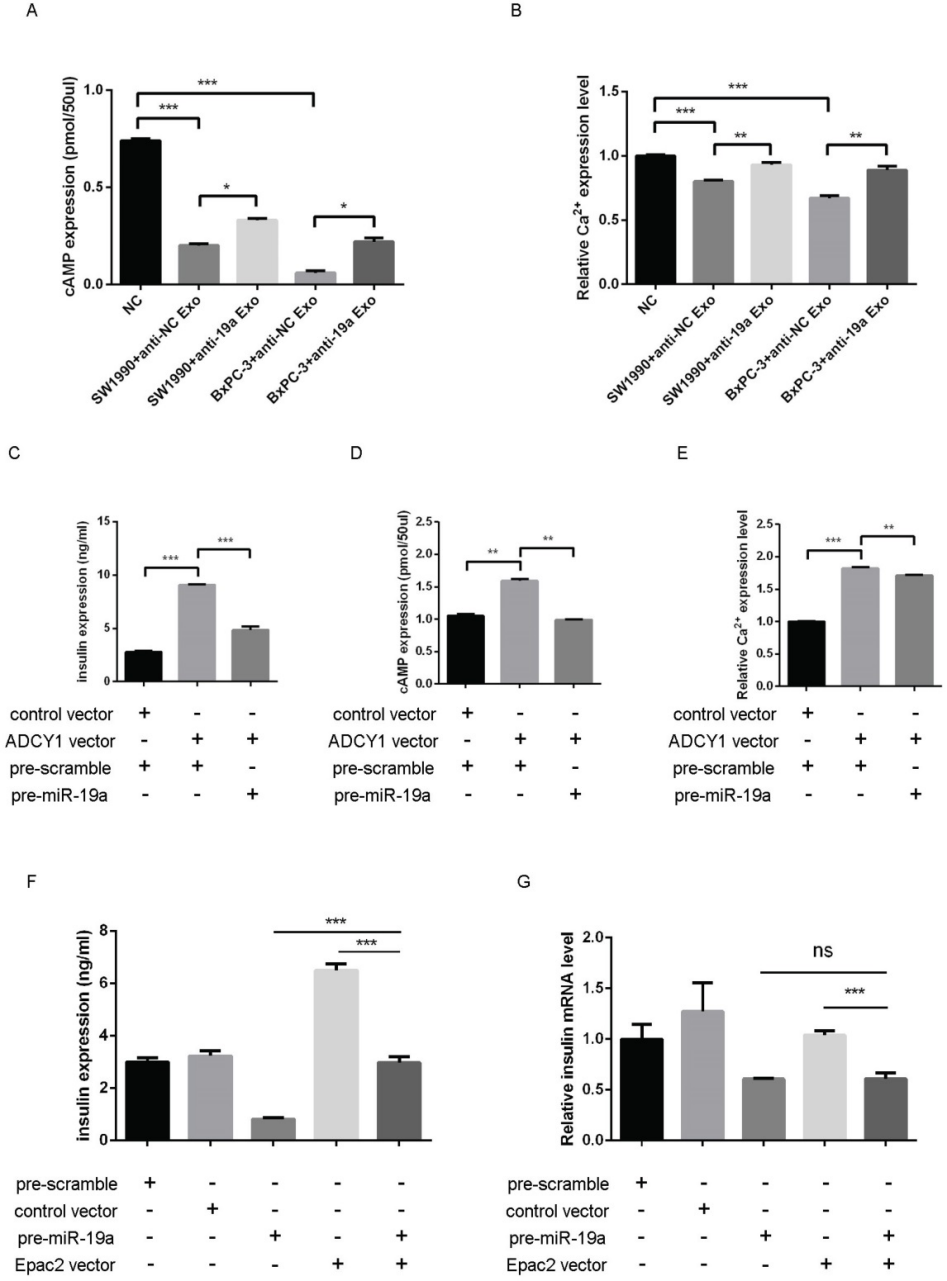
** (A)** cAMP expression level in MIN6 cells treated with the miR-19a-deficient PaC exosomes and control PaC exosomes. **(B)** Ca^2+^ concentration in MIN6 cells treated with miR-19a-deficient PaC exosomes and control PaC exosomes. **(C)** GSIS assay in MIN6 cells co-transfected with pre-miR-19a, Adcy1 OE plasmid, and corresponding controls. **(D)** cAMP expression level in MIN6 cells co-transfected with pre-miR-19a, Adcy1 OE plasmid, and corresponding controls. **(E)** Ca^2+^ concentration in MIN6 cells co-transfected with pre-miR-19a, Adcy1 OE plasmid, and corresponding controls. **(F)** GSIS assay in MIN6 cells co-transfected with pre-miR-19a, Epac2 OE plasmid, and corresponding controls. **(G)** Insulin mRNA expression levels in MIN6 cells co-transfected with pre-miR-19a, Epac2 OE plasmid, and corresponding controls. *P < 0.05; **P < 0.01; ***P < 0.001.

Whether the exosomal miR-19a and the target *Adcy1* and *Epac2* genes altered the levels of cAMP and Ca^2+^ remained to be examined. The SW1990 and BxPC-3 exosomes with or without pretreatment with anti-miR-19a were prepared. MIN6 cells treated with control PaC exosomes presented even lower cAMP expression compared with those treated with miR-19a-deficient PaC exosomes (Fig. 5A). Also, the [Ca^2+^]i showed parallel alteration in these different groups (Fig. 5B). Based on these results, it was concluded that PaC exosomal miR-19a was involved in the GSIS, but it was not the only way that impelled the GSIS.

Then, the impact of miR-19a and Adcy1 on insulin, cAMP, and [Ca^2+^]i was assessed separately. The transfection of the Adcy1 overexpressing plasmid might upregulate insulin, cAMP, and [Ca^2+^]i, and this effect was declined by introduced pre-miR-19a to some extent (Fig. 5C-E). The study demonstrated that overexpressed Epac2 might increase the insulin secretion level, and this effect was partly weakened by pre-miR-19a (Fig. 5F); whereas, the insulin mRNA level was not disturbed by the additional Epac2 (Fig. 5G).

### Implications of miR-19a for the impairment of GSIS in PaC homograft mice

**Figure 6:**
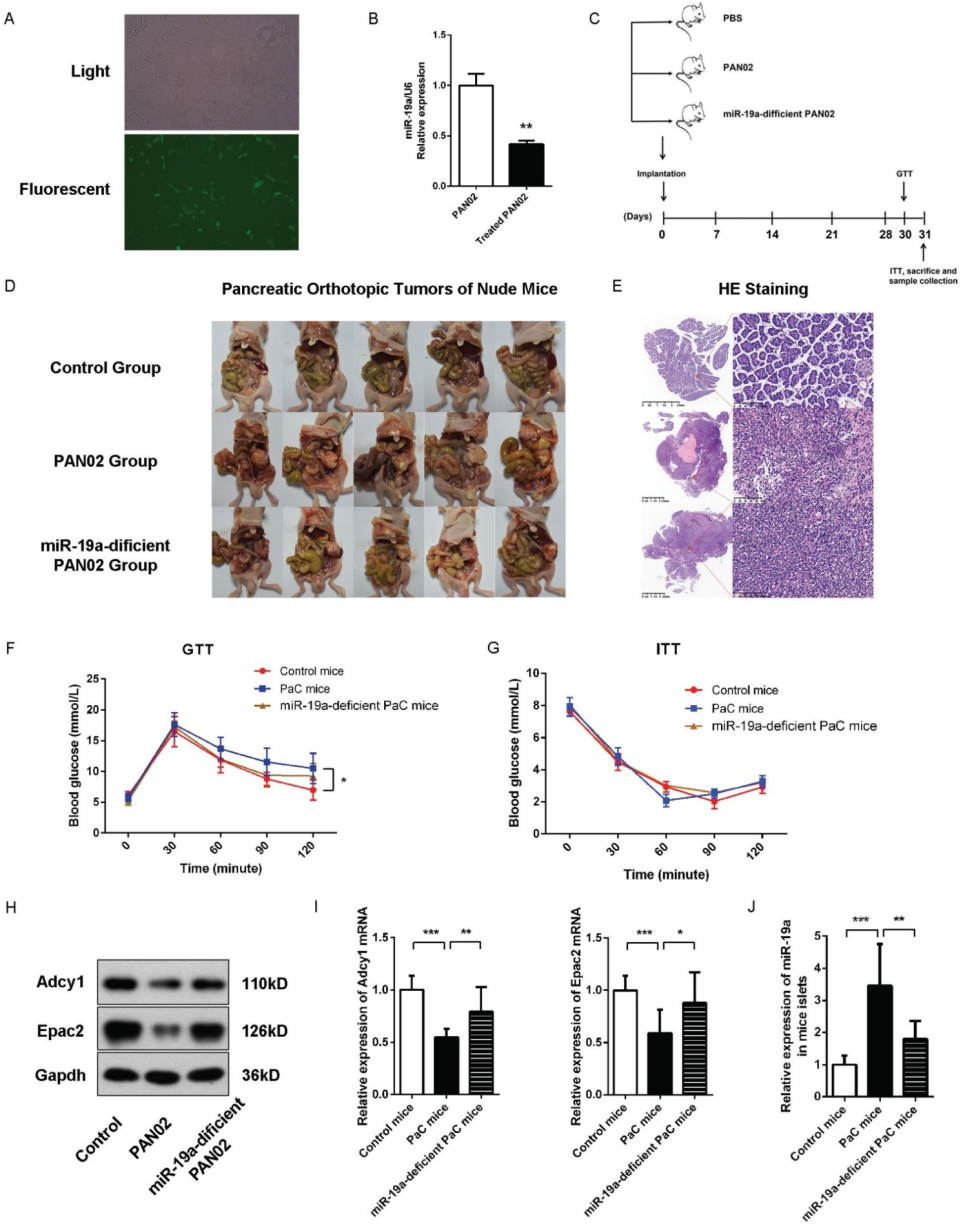
** (A)** Representative image of the PAN02 cells stably transfected by fluorescence-labelled lentivirus. **(B)** Quantitative analysis of miR-19a between PAN02 and lentivirus-treated PAN02. **(C)** Flow chart of the *in vivo* experiment design. Nude mice were first implanted orthotopically with miR-19a-deficient PAN02, untreated PAN02 (2.5×10^6^ cells per mouse) or PBS as controls. Then, each mouse was inspected every week to 30 days. Before mice sacrifice and samples collection, IPGTT and ITT tests were performed in 2 separated days. **(D)** The images of the orthotopic tumors. **(E)** The verification of the orthotopic tumors by HE staining. **(F)** IPGTT tests. **(G)** ITT tests. **(H)** Western blot analysis of Adcy1 and Epac2 protein levels in primary islets of the 3 groups. **(I)** Relative Adcy1 and Epac2 mRNA expression levels in primary islets of the 3 groups. **(J)** Relative miR-19a expression levels in primary islets of the 3 groups. *P < 0.05; **P < 0.01; ***P < 0.001.

Subsequently, we evaluated the effects of PaC-induced glycometabolic disorders *in vivo* and the role of miR-19a in GSIS. We generated a stable miR-19a-knock-down PAN02 cell line with the GFP-labelled lentivirus (Fig. 6A-B). Nude mice orthotopic transplantation models were constructed by the injection of 2.5×106 generated stable miR-19a-deficient PAN02 cells or the equivalent original PAN02 cells in 50ul PBS per mouse, and 50 ul PBS per mouse in control group (5 mice per group). Before homograft mice were sacrificed 30 days after PaC cells implantation, the intraperitoneal glucose tolerance test (IPGTT) and insulin tolerance test (ITT) were tested at 30 and 31 days after homograft, respectively, before the sacrifice of mice and samples collection (Fig. 6C). The homograft pancreatic cancers were confirmed by HE staining (Fig. 6D-E). As shown in Fig. 6F and 6G, the PaC mice groups showed significant augmented glucose levels at 90 and 120 min in IPGTT comparing to control group, while no significant differences of glucose levels were observed among three groups in ITT. On the other hand, the miR-19a-defficient PaC group showed less impaired glucose intolerance, comparing to the untreated PaC group. Thus, we could conclude that the PaC homograft mice demonstrated the reduction of insulin secretion after high glucose stimulation, but no dramatic sign of peripheral insulin resistance, while the pancreatic derived miR-19a plays an important role. Furthermore, after the isolation of the islets, we measured the studied protein levels in mice islets. Compared to control mice group, the Adcy1 and Epac2 protein levels as well as the mRNA levels were lower in PAN02 group but partially regained in miR-19a-deficient PAN02 group (Fig. 6H and 6I). Parallelly, the islet miR-19a levels were upregulated in PaC mice group (Fig. 6J), which corresponded to our hypothesis.

## Discussion

Previous studies showed that supernatants from pancreatic cancer cell lines induced β-cell dysfunction *in vivo* and *in vitro*. However, the exact mechanism how PaC cells induced β cells remained unclear 28. Pancreatic islet β-cells release insulin to lower the glucose level in response to hyperglycemia; dysfunction of this process might account for the pathological development of glycometabolic disorders secondary to PaC 29. The cyclic adenosine monophosphate (cAMP), produced by cell membrane protein adenylyl cyclase (ADCY) and ATP, potentiates insulin secretion in both PKA-dependent and PKA-independent manners, which could be affected by ADCY activators or inhibitors 30. The ADCY family comprised 10 isoforms, and the mammalian Adcy1 is supposed to be a critical enzyme activated by extracellular Ca^2+^ in MIN6 cells. The silencing of endogenous Adcy1 by siRNA significantly suppressed the changes in the second messengers [cAMP]i and [Ca2+]i after the application of glucose 31. On the contrary, Seino's group 32, 33 demonstrated that the cAMP-binding protein cAMP-GEFII, also known as Epac2, mediated cAMP-dependent, PKA-independent exocytosis in incretin-potentiated insulin secretion in pancreatic β cells. The present study reported that PaC-derived exosomes could induce a defect of GSIS, and exosomal miR-19a might be a pivotal mediator by targeting directly the two pivotal molecules, ADCY1 and EPAC2, and contributing into the pathogenesis of PaC-DM. This study was novel in reporting the dysregulation of miR-19a in MIN6 cells after the stimulation by PaC cells; transfection with pre-miR-19a could induce a defect of the GSIS. It was suggested that the secretion of miR-19a targeting Adcy1 and Epac2 was one of the mechanisms by which miR-19a disturbed glycometabolism. Then, miR-19a-deficient exosomes from PaC cells were used to confirm that the major effect of exosomes on MIN6 cells was due to miR-19a rather than many other potential constitutions in the exosomes. A previous study showed the levels of endogenous miRNAs did not substantially change after the induction of exogenous exosomes. Also, negligible changes in protein/mRNA/miRNA levels in miRNA antisense-transfected exosomes were found, revealing the use of miR-19a-deficient exosomes in the present study 34. Meanwhile, the elevation of the miR-19a levels but not the pre-miR-19a levels in MIN6 cells, after stimulated by PaC exosomes, demonstrated that the alteration of miR-19a in MIN6 was exogenous rather than endogenous (Fig. S2A). Thus, this study revealed at least one of the important mechanisms by which PaC cells influenced adjacent β cells.

In PaC-DM, various research suggested that both β-cell dysfunction and increased peripheric insulin resistance engaged in the etiopathogenesis 8, 29. Usually, an increased insulin exocytosis secondary to peripheric insulin resistance maintains the glycometabolic stability. But our data demonstrated that there might be improper response for this physiological reaction due to the effect by secreted factors by PaC cells, which promote the destabilization of glucose homeostasis. A recent study showed that adrenomedullin (ADM) was upregulated in patients with pancreatic cancer and caused insulin resistance in β cells and mice 8, and was subsequently found in pancreatic exosomes 35. Whether the exosomal miR-19a and ADM contribute together, or interplay need further investigations.

The study found an obvious influence of GSIS with the exosomes from SW1990 and BxPC-3 cells, whereas only a slight change was observed with the supernatants. The disagreement was probably because miR-19a was much more enriched in exosomes than in supernatants. In addition, the study also used another PaC cell line PANC1 and showed that the supernatant or exosomes from PANC1 cells could only feebly disrupt the GSIS, although the miR-19a-targeting protein levels apparently changed (data not shown). These findings suggested that the pathogenesis of PaC-DM was a complex disorder, and exosome-enclosed miRNAs represented a heterogeneous population involving other pathogenic mechanisms. Moreover, the study also found downregulated insulin mRNA levels after the transfection of pre-miR-19a (Fig. 5G); it focused on the potential targets of miR-19a in reducing insulin production, which have recently been validated by our group (data not shown). Therefore, the investigation simply described one of the major pathways involved in the dysregulation of insulin exocytosis.

However, this study had some limitations. The nucleotides in seed recognition sites of Adcy1 and Epac2 3'-UTR and miR-19a are totally conserved across species, including human and mouse (Supplementary Fig. 2D), which made the regulation of mouse genes by miRNA from human cell lines reasonable. Also, emerging studies confirmed the biological effect of miRNAs in cross-species communication and cross-kingdom regulation 36-40. Hence, β cell line MIN6 was used as the model in the present study.

Exclusive bidirectional interaction between pancreatic cancer and new-onset DM (commonly considered as less than 3 years) might guide the design of potential approaches for the early diagnosis of PaC. The ultimate of the research by our group are to identify the more specific underlying biomarkers to distinguish early stage PaC patients from the enormous population of T2DM. Thus, more comprehending of the mechanism of PaC-DM helps for formulating strategies. Taken together, our studies provide new insight into molecular mechanism of the paraneoplastic diabetes in PC. Further studies are required for deeper understanding of this physiopathology and explore potential screening tools.

## Supplementary Material

Supplementary methods, figures and tables.
