# Supplementary material for: Pancreatic cancer-derived exosomal microRNA-19a induces β-cell dysfunction by targeting ADCY1 and EPAC2
Source: Int J Biol Sci. 2021 Aug 21;17(13):3622–33. doi: 10.7150/ijbs.56271 (PMC8416731; doi:10.7150/ijbs.56271)
Supplement: Supplementary file 1 — Supplementary methods, figures and tables. [file ijbsv17p3622s1.pdf]

SUPPLEMENTARY MATERIALS

Supplementary Methods

Measurement of glucose-stimulated insulin secretion

The cells or islets were plated in 24-well poly-D-lysine-coated plates at a density of ~3 × 10^5 cells/well or 10 size-matched islets/well. The media were removed, and the cells or islets were washed once and then incubated for 2 h in modified Krebs–Ringer medium (125 mmol/L NaCl, 4.74 mmol/L KCl, 1 mmol/L $CaCl_2$, 1.2 mmol/L KH_2PO_4, 1.2 mmol/L $MgSO_4$, 5 mmol/L $NaHCO_3$, and 25 mmol/L 2-[4-(2-Hydroxyethyl)-1-piperazinyl]ethanesulfonic acid (HEPES), pH 7.4) containing 0.1% bovine serum albumin (BSA) and 2.8 mmol/L glucose 48 h after transfection or stimulation by cancer cells. The cells or islets were then subjected to 28 mmol/L glucose treatment for 1 h before the supernatant fraction was removed for insulin determination.

Fluorescence labeling of exosomes

Briefly, SW1990 and BxPC-3 cells were labeled with DiI-C16 (Beyotime, China) for 1 h and then washed three times with PBS. The cells were resuspended and cultured overnight in DMEM medium supplemented with 10% FBS. The supernatants were then collected to harvest exosomes. The exosomes were resuspended in DMEM medium and incubated with cultured MIN6 cells. After incubation for 12 h, the MIN6 cells were washed, fixed, and observed. MIN6 cells treated with normal PaC-derived exosomes served as a negative control.

Plasmid construction and luciferase assay

The partial 3’-untranslated regions (3’-UTRs) of human *Adcy1* and *Epac2*, which included the predicted miR-19a-binding sites, were synthesized with an additional 3'-phosphorylation modification. For luciferase reporters containing mutant *Adcy1* and *Epac2* 3’-UTRs, the sequences that interacted with bases 2 – 8 of the miR-19a seed sequence were mutated and inserted into equivalent luciferase reporters.

For luciferase reporter assays, the cells were cultured in 24-well plates and transfected with 2 μg firefly luciferase reporter plasmid, 2 μg β-galactosidase expression plasmid (Ambion, ThermoFisher Scientific, USA), and equal amounts of scrambled negative control RNA, pre-miR-19a, or anti-miR-19a using Lipofectamine 2000. The assays were performed using luciferase assay kits (Promega, USA) 24 h after transfection.

Glucose-induced Ca^{2+} oscillation imaging in cultured β-cells

After the MIN6 cells were switched to 28mM glucose during acquisition, the cells were alternately illuminated at 340 and 380 nm, whereas fluorescence emission was recorded at 519 nm. The results of Fura-2/AM were indicated by the ratio of F340/F380.

Supplementary Table1.

Primer Names	Primer Sequence (5’→3’)
m-Adcy1-F	GTTCACAGC AGACACGATG
m-Adcy1-R	GGCACTGGTTGA CTATGTA
m-Epac2-F	CAAGGAGAATGTCCCTTCAGAGA
m-Epac2-R	CCGCGAGTGAACACAGGAT
m-Insulin-F	GTGGGGGCTCGTGTTTCTC
m-Insulin-R	GATCACCGTGCAGTTTTCCA
m-pre-miR-19a-F	GCAGCCCTCTGTTAGTTTTGC
m-pre-miR-19a-R	CAGGCCACCATCAGTTTTGC
m-Gapdh-F	AGGTCGGTGTGAACGGATTTG
m-Gapdh-R	GGGGTCGTTGATGGCAACA
m-β-actin-F	AGGGAAATCGTGCGTGAC
m-β-actin-R	CGCTCATTGCCGATAGTG
h-miR-19a	TGTGCAAATCTATGCAAAACTGA
h-U6-F	CTCGCTTCGGCAGCACA
h-U6-R	AACGCTTCACGAATTTGCGT

Supplement Figures:

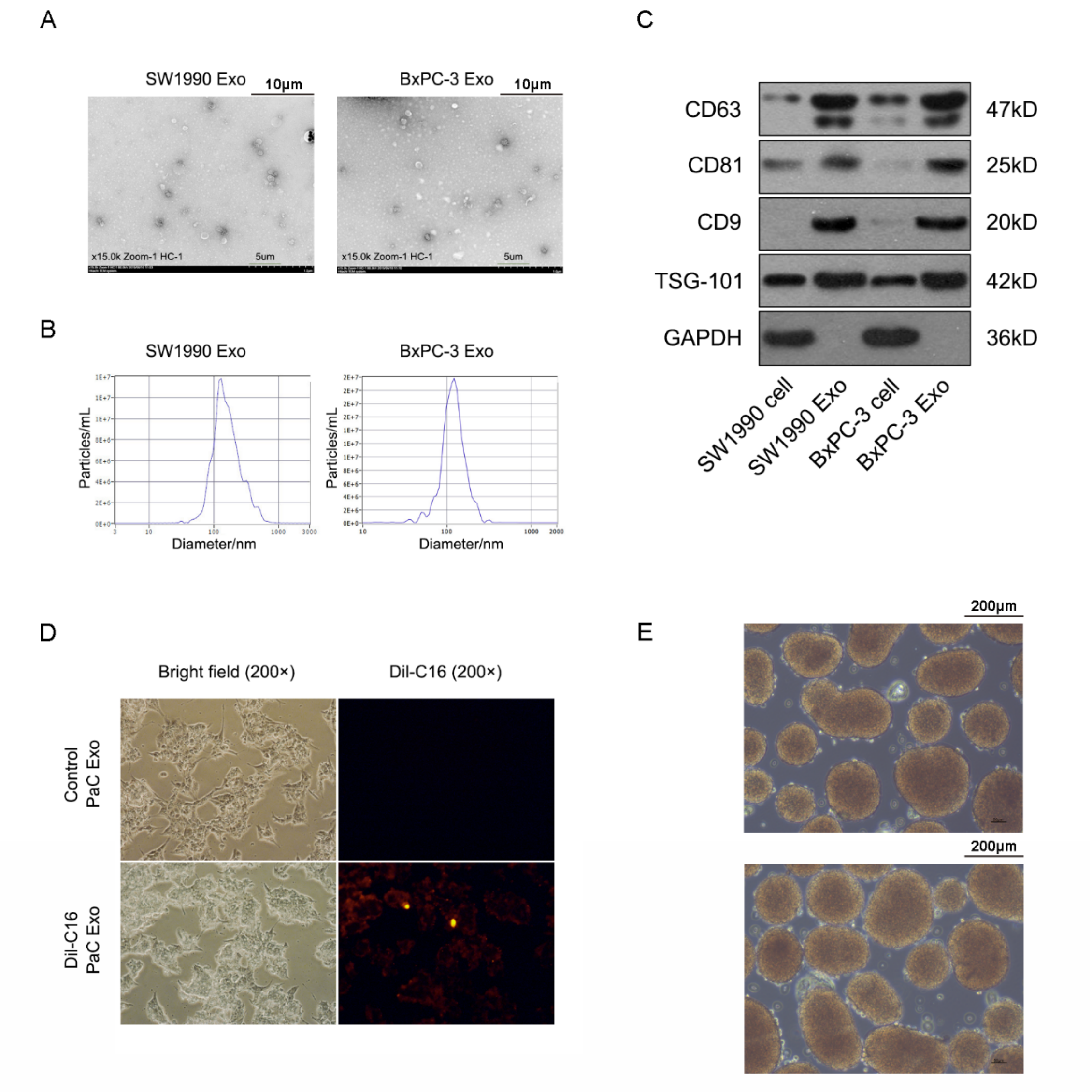

Supplementary Figure 1. (A) Typical transmission electron microscopic images of SW1990 and BxPC-3 exosomes. (B) Size distributions of exosomes isolated from different cancer cells by NTA. (C) Levels of the exosomal marker proteins CD63, CD81, CD9, and TSG101 in cell lysates and cell exosomes were determined using Western blot analysis. GAPDH was selected as a negative marker. (D) Incorporation of PaC cell exosomes into MIN6 cells. (E) Isolated primary mouse islets.

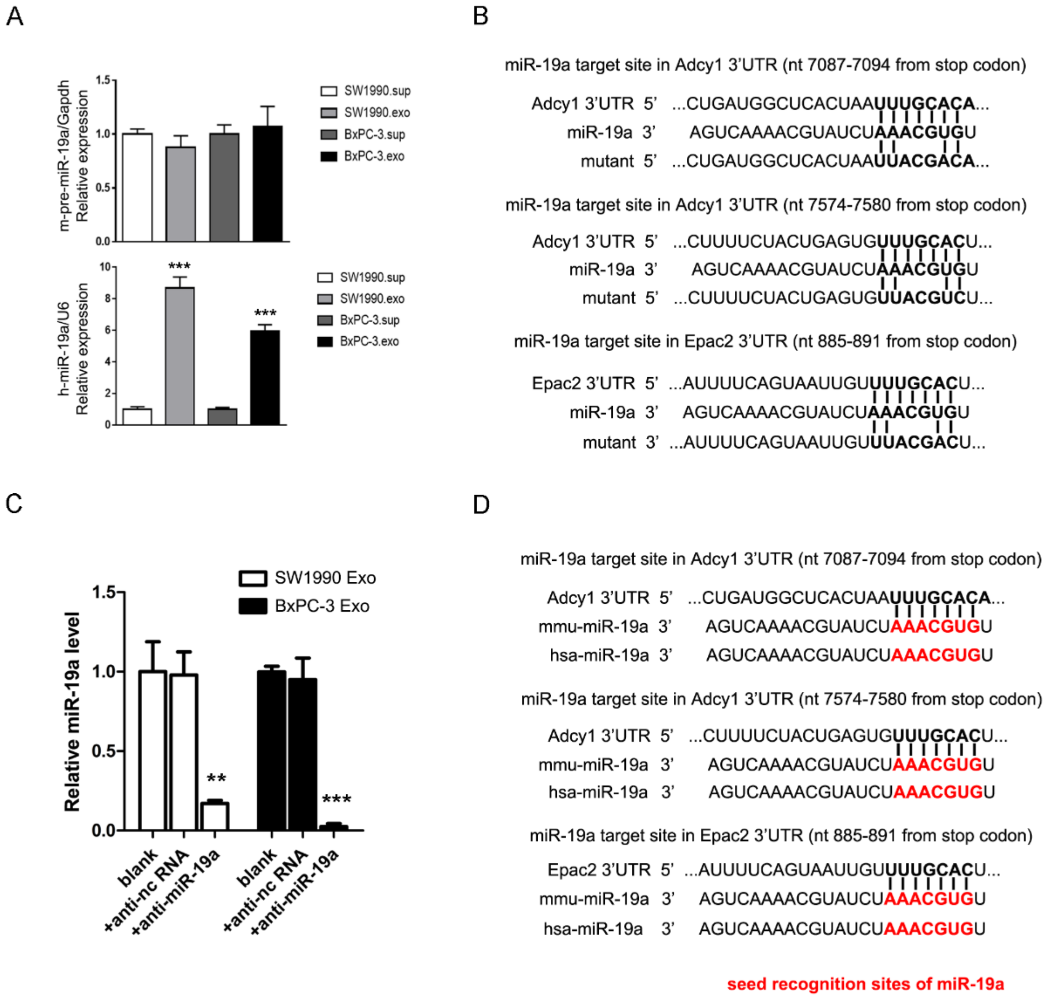

Supplementary Figure 2. (A) Pre-miR-19a and miR-19a mRNA levels in MIN6 cells treated with PaC cell supernatants and exosomes. (B) Wild-type (wt) or mutant (mut) miR-19a-binding sites (the sequences that interacted with two to eight bases of miR-19a were mutated) in Adcy1 or Epac2 3'-UTR are depicted. (C) Knockdown efficiency of anti-miR-19a in exosomes from SW1990 and BxPC-3 cells. (D) Schematic description of the hypothetical duplexes formed by the interactions between the binding sites in Adcy1 and Epac2 3'-UTR and miR-19a. The seed recognition sites are denoted in red, and all nucleotides in these regions are totally conserved across humans and mice. *$P < 0.05$; **$P < 0.01$; ***$P < 0.001$.
